# Supplementary material for: Curriculum-based outdoor learning for children aged 9-11: A qualitative analysis of pupils’ and teachers’ views
Source: PLoS One. 2019 May 31;14(5):e0212242. doi: 10.1371/journal.pone.0212242 (PMC6544203; doi:10.1371/journal.pone.0212242)
Supplement: S2 Appendix — (DOCX) [file pone.0212242.s002.docx]

# S2 Appendix. Focus Group and Interview Topic Guides.

Focus Group Topic Guide (Pupils)

Baseline (January)

Brief introduction

1. Can you tell me what you know about outdoor learning? Prompt: Have you done any learning outdoors in the past?
2. What do you think about learning outdoors? Prompts: what do you think is good about it? What do you think is bad about it?
3. Do you think being outdoors makes any difference to how you and the people in your class learn or behave? In what way??
4. What does everyone else in your class think about outdoor learning?
5. What are you looking forward to? Is there anything you are not looking forward to?
6. Can you tell me if you think there will be any problems with learning outside? Do you have any suggestions of how to make it work well?
7. Do you think learning outdoors will change how well you do in school and in what way?
8. Do you have anything else to add about your thoughts about this project in general?

Focus Group Topic Guide (Pupils)

Follow up (July)

Brief introduction

1. What did you think about learning outdoors? Prompts: what did you think was good about it? What do you think was bad about it?
2. What did everyone else in your class think about outdoor learning?
3. Do you think being outdoors made any difference to how you and the people in your class learnt or behaved? In what way??
4. Can you tell me if there were there any problems with learning outside? Do you have any suggestions of how to make it work better in the future?
5. Do you think learning outdoors is changing how well you are doing in school and in what way?
6. Would you like to carry on with learning outdoors? Prompts: Does everyone in your class want to carry on with learning outdoors?
7. Can you tell me if you think other schools should do learning outdoors and why??
8. Do you have anything else to add about your thoughts about this project in general?

Interview Topic Guide (Teachers)

Baseline (January)

Brief introduction

1. What do you know about outdoor learning? Prompt: Have you or your pupils done any learning outdoors in the past?
2. How do you feel about delivering outdoor learning to your class? Do you feel confident in teaching outdoors? If not, why?
3. Can you tell me whether you think being outdoors will make any difference to how your pupils learn or behave? Or any other effects?
4. Can you tell me whether you think learning outdoors will change how well your pupils do in school and in what way?
5. Do you think there will be any problems with learning outside? Do you have any suggestions of how to make it work well?
6. How do your pupils feel about taking part in outdoor learning? Prompts: What are they looking forward to? What are they not looking forward to?
7. Do you have anything else to add about your thoughts about this project in general?

Interview Topic Guide (Teachers)

Follow up (July)

Brief introduction

1. Can you tell me about how the outdoor learning programme went? Prompt: How was it different to teaching indoors?
2. How did you feel about delivering outdoor learning to your class? Did you feel confident in teaching outdoors? If not, why? Would you feel confident in teaching another outdoor learning project now?
3. Did you feel supported throughout the delivery of the project? Did you receive any training to deliver it?
4. Can you tell me whether you think being outdoors made any difference to how your pupils learnt or behaved? Or any other effects?
5. Can you tell me whether you think learning outdoors changed how well your pupils were doing in school and in what way?
6. Do you think there were any problems with learning outside? Do you have any suggestions of how to make it work better if other schools were to deliver the project?
7. How did your pupils feel about taking part in outdoor learning?
8. Would you like to continue teaching the outdoor learning programme? Do you think other schools should deliver outdoor learning? Why/why not?
9. Do you have anything else to add about your thoughts about this project in general?

Interview Topic Guide (Head teachers)

Follow up (July)

1. Can you tell me why you first became interested for your school to take part in outdoor learning? What were the main outcomes you were interested in?
2. Can you tell me how the outdoor learning programme went? What went well, didn’t go so well?
3. How did your school deliver outdoor learning? Where, how often?
4. How did your staff feel about delivering outdoor learning? Did they receive training?
5. How did pupils feel about taking part in outdoor learning?
6. Can you tell me whether you think outdoor learning made any difference to pupils?
7. Have you had any feedback from pupils/parents/staff?
8. Do you think there were any problems with outdoor learning? Do you have any suggestions of how to make it work better if other schools were to deliver the project?
9. Would you like/will your school continue outdoor learning?
10. Do you think other schools should deliver outdoor learning? Why/why not? Any recommendations?
11. Do you have anything else to add about your thoughts about this project in general?
