# Supplementary material for: Curriculum-based outdoor learning for children aged 9-11: A qualitative analysis of pupils’ and teachers’ views
Source: PLoS One. 2019 May 31;14(5):e0212242. doi: 10.1371/journal.pone.0212242 (PMC6544203; doi:10.1371/journal.pone.0212242)
Supplement: S3 Appendix — (DOCX) [file pone.0212242.s003.docx]

| **Theme** | **Sub-Theme** |
| --- | --- |
| Expectations and experience of outdoor learning | Feeling free |
|  | Exposure to environment and safety |
|  | Pupil engagement |
| Factors influencing outdoor learning | Motivations |
|  | Curriculum pressure and accountability |
|  | Natural resources |
|  | Physical resources |
|  | Support |
|  | Teacher influence |
| Perceived impact on learning and development | Behaviour |
|  | Concentration and memory |
|  | Key skills development |
|  | Health and wellbeing |

# S3 Appendix. Themes and Sub-themes.
